# Preface to Special Issue on ‘Cytochrome P450 Variation in Pharmacogenomics’

**DOI:** 10.3390/jpm8030023

**Published:** 2018-07-04

**Authors:** Allan E. Rettie, Stephen B. Liggett

**Affiliations:** 1 Department of Medicinal Chemistry, University of Washington, Seattle, WA 98195, USA; 2 Department of Internal Medicine and Molecular Pharmacology and Physiology, and the Center for Personalized Medicine and Genomics, University of South Florida Morsani College of Medicine, Tampa, FL 33612, USA; sliggett@health.usf.edu

Patient-to-patient variability in response to drugs creates a significant challenge for the safe and effective treatment of many human diseases. Pharmacogenomics seeks to address this challenge by linking drug response to patient genotypes at important loci in order to better customize patient treatments. While many diverse ‘pharmacogenes’ are recognized (e.g., *TPMT*, *G6PD*, *HLA*, etc.), a thorough understanding of the implications of genetic variation in the *CYP* superfamily, especially, provides a golden opportunity to practice precision medicine and improve therapeutic outcomes. Rare variation in the steroidogenic cytochrome P450 (CYP) enzymes has long been associated with some inborn errors of metabolism [1]. However, it is an appreciation of the consequences of more commonly occurring variation in the drug-metabolizing CYPs—especially the large *CYP2* gene family—that has been a driver in the development of contemporary pharmacogenomics.

The importance of CYP-based pharmacogenomics can be gleaned from Figure 1 below, which depicts the relative percentages of the most frequently identified genes/gene families on the Clinical Pharmacogenetics and Implementation Consortium (CPIC) prioritized drug–gene pairs list (https://cpicpgx.org/genes-drugs/). CPIC identifies Level A or B drug–gene pairs as those where prescribing action based on pharmacogenetic information is recommended. Of the 153 Level A or B gene pairs (accessed February 2018), there were 106 instances where the gene features in an interaction with more than two prescription drugs. Within this subset, *CYP* genes constituted 50% of the entries. The next most important gene/gene family is *G6PD*, followed distantly by *HLA* genes.

Worldwide, the distribution of *CYP* alleles varies substantially between populations, with attendant consequences for global health [2]. *CYP* variation, and its functional effects on enzyme activity (where known), is cataloged at the PharmVar web-site (https://www.pharmvar.org/). This repository continues to grow and to be a valuable resource to inform clinical decision-making [3].

Against this backdrop, it felt particularly timely to devote a Special Issue of the *Journal of Personalized Medicine* to the topic of ‘Cytochrome P450 Variation in Pharmacogenomics’. This issue, which comprises eight original articles, begins by reviewing the fundamental biochemistry, genetics and clinical importance of CYP2A6 [4], CYP2C9 [5] CYP2C19 [6] and CYP2D6 [7]. This is followed with an article on translational applications of *CYP* pharmacogenomics in guiding tamoxifen and warfarin therapy in Canada [8] and two papers on CYP pharmacogenomics in special populations [9,10]. The issue closes with a commentary on the future of warfarin pharmacogenomics, long heralded as the ‘poster-child’ of pharmacogenomics [11].

We hope this collection of papers will prove a useful introduction to CYP pharmacogenetics at large, provide an update on contemporary translational efforts in the clinic and stimulate further basic research in the field.

## Figures and Tables

**Figure 1:**
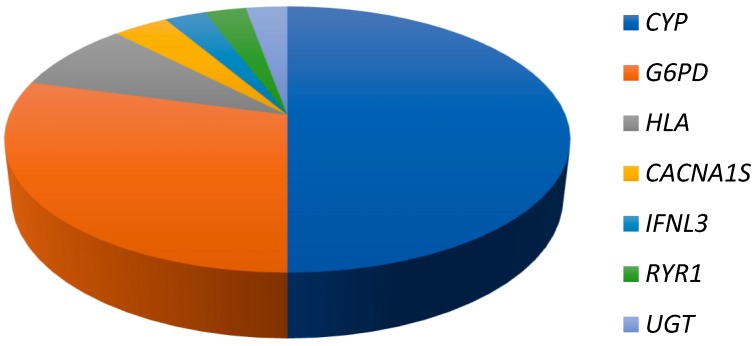
Importance of CYP pharmacogenomics. The pie chart depicts the percentage that each set of pharmacogenes contributes in priority drug–gene pair interactions with two or more drugs.
